# Factores asociados al cribado de Diabetes Mellitus en población Peruana ¿problema para la salud pública?*

**DOI:** 10.15649/cuidarte.2792

**Published:** 2023-05-28

**Authors:** Fiorella Trujillo-Minaya, Víctor Juan Vera-Ponce, Jenny Raquel Torres-Malca, Fiorella E. Zuzunaga-Montoya, Jamee Guerra-Valencia, Jhony A. De-La-Cruz-Vargas, Liliana Cruz-Ausejo

**Affiliations:** 1 . Instituto de Investigaciones en Ciencias Biomédicas, Universidad Ricardo Palma, Lima, Perú. Email: fiorella.trujillo@urp.edu.pe Universidad Ricardo Palma Instituto de Investigaciones en Ciencias Biomédicas Universidad Ricardo Palma Lima Peru fiorella.trujillo@urp.edu.pe; 2 . Instituto de Investigaciones en Ciencias Biomédicas, Universidad Ricardo Palma, Lima, Perú. Email: vicvepo@gmail.com Universidad Ricardo Palma Instituto de Investigaciones en Ciencias Biomédicas Universidad Ricardo Palma Lima Peru vicvepo@gmail.com; 3 . Instituto de Investigaciones en Ciencias Biomédicas, Universidad Ricardo Palma, Lima, Perú. Universidad Tecnológica del Perú, Lima, Perú. Email: ylsa2@hotmail.com Universidad Ricardo Palma Instituto de Investigaciones en Ciencias Biomédicas Universidad Ricardo Palma Lima Peru ylsa2@hotmail.com; 4 . Instituto de Investigaciones en Ciencias Biomédicas, Universidad Ricardo Palma, Lima, Perú. Email: fiorellazuzunaga@gmail.com Universidad Ricardo Palma Instituto de Investigaciones en Ciencias Biomédicas Universidad Ricardo Palma Lima Peru fiorellazuzunaga@gmail.com; 5 . Universidad Científica del Sur, Lima, Perú. Email: jamee.guerra.valencia@gmail.com Universidad Científica del Sur Universidad Científica del Sur Lima Peru jamee.guerra.valencia@gmail.com; 6 . Instituto de Investigaciones en Ciencias Biomédicas, Universidad Ricardo Palma, Lima, Perú. Email: jadv.oncology@gmail.com Universidad Ricardo Palma Instituto de Investigaciones en Ciencias Biomédicas Universidad Ricardo Palma Lima Peru jadv.oncology@gmail.com; 7 . Universidad Científica del Sur, Lima, Perú. Email: rcruzausejo@gmail.com Universidad Científica del Sur Universidad Científica del Sur Lima Peru rcruzausejo@gmail.com

**Keywords:** Diabetes Mellitus, Tamizaje Masivo, Factores Epidemiológicos, Perú, Diabetes Mellitus, Mass Screening, Epidemiologic Factors, Peru, Diabetes Mellitus, Programas de Rastreamento, Fatores epidemiológicos, Perú

## Abstract

**Introducción:**

La Diabetes Mellitus tipo 2 es una enfermedad que representa un reto para la salud pública por su tendencia al crecimiento e impacto sobre todo en países en desarrollo.

**Objetivo:**

determinar los factores asociados a la no realización del cribado de diabetes mellitus tipo 2 según la encuesta demográfica y de salud familiar del año 2020 (ENDES-2020).

**Materiales y métodos:**

Estudio analítico transversal secundario de la ENDES-2020.

**Resultados:**

Las variables que mostraron asociación estadísticamente significativa para cribado de DM2 fueron: sexo masculino (PR=1,06, IC95% 1,02-1,10; p<0,001), edad entre 30 a 59 años (0,92; IC95% 0,89-0,95; p<0,001) y 60 años a más (PR=0,72; IC95% 0,65-0,79; p<0,001), educación primaria (PR=0,94, IC 95% 0,92 - 0,99; p<0,020), secundaria (PR=0,93; IC 95% 0,88-0,97; p=0,008) y superior (PR=0,86, IC 95% 0,85-0,94; p<0,001), ser pobre (PR=0,96, IC95% 0,92-0,99; p=0,016), medio (PR=0,93; IC95% 0,88 - 0,96; p=0,001), rico (PR=0,89; IC95% 0,84 - 0,94; p<0,001), muy rico (PR=0,81; IC95% 0,75-0,86; p<0,001), e hipertensión (PR=0,91; IC 95% 0,867-0,969; p=0,002).

**Discusión:**

El sexo masculino fue el único factor asociado a la no realización del cribado de diabetes mellitus tipo 2, mientras que, pertenecer a un grupo de edad mayor, tener hipertensión arterial, mayor nivel educativo y socioeconómico aumentó la posibilidad de realizarlo.

**Conclusión:**

Es imprescindible reforzar las estrategias de cribado en el primer nivel de atención, mediante la implementación de medidas de prevención.

## Abstract (en)

**Introduction:**

Type 2 Diabetes Mellitus (T2DM) is a disease that poses a challenge to public health due to its growth trend and impact, especially in developing countries. This study aimed to determine the factors associated with not being screened for T2DM according to the 2020 Demographic and Family Health Survey (ENDES-2020).

**Materials and Methods:**

Secondary, cross-sectional, analytical study using the ENDES-2020 data.

**Results:**

The variables that showed a statistically significant association for T2DM screening were the following: male sex (PR=1.06, 95% CI: 1.02-1.10; p<0.001), ages between 30 and 59 years (0.92, 95% CI: 0.89-0.95; p<0.001) and 60 years and older (PR=0.72, 95% CI: 0.65-0.79; p<0.001), primary education (PR=0.94, 95% CI: 0.92-0.99; p<0.020), secondary education (PR=0.93, 95% CI: 0.88-0.97; p=0.008) higher education (PR=0.86, 95% CI: 0.85-0.94; p<0.001), poor (PR=0.96, 95% CI: 0.92-0.99; p=0.016), middle income (PR=0.93, 95% CI: 0.88-0.96; p=0.001), rich (PR=0.89, 95% CI: 0.84-0.94; p<0.001), very rich (PR=0.81, 95% CI: 0.75-0.86; p<0.001), and hypertension (PR=0.91, 95% CI: 0.867-0.969; p=0.002).

**Discussion:**

Being male was the only factor associated with not being screened for T2DM, whereas belonging to an older age group, having arterial hypertension, and having higher education and socioeconomic levels increased the possibility of being screened.

**Conclusion:**

It is essential to strengthening screening strategies at the primary level of care by implementing preventive measures.

## Resumo

**Introdugáo:**

A diabetes melito tipo 2 é uma doenga que representa um desafio a saúde pública devido a sua crescente tendencia e impacto, especialmente em países em desenvolvimento. O objetivo deste estudo foi determinar os fatores associados a nao realizagao de triagem para diabetes mellitus tipo 2 de acordo com a Pesquisa Demográfica e de Saúde da Família 2020 (DHS-2020).

**Materiais e métodos:**

Estudo analítico transversal secundário do ENDES-2020.

**Resultados:**

As variáveis que mostraram associagao estatisticamente significativa para a triagem DM2 foram: sexo masculino (PR=1,06, 95%CI 1,02-1,10, p<0,001), idade 30-59 anos (0,92, 95%CI 0,89-0,95, p<0,001) e 60 anos ou mais (PR=0,72, 95%CI 0,65-0,79, p<0,001), educagao primária (PR=0,94, 95%CI 0,92-0,99, p<0,020), educagao secundária (PR=0,93, 95%CI 0,88-0,97, p=0,008), e educagao secundária superior (PR=0,93, 95%CI 0,88 0,97, p=0,008); p=0,008) e superior (PR=0,86, 95% CI 0,85-0,94; p<0,001), pobre (PR=0,96, 95% CI 0,92 0,99; p=0,016), médio (PR=0,93, 95% CI 0,88 - 0,96; p=0,001), rica (PR=0,89; 95%CI 0,84 - 0,94; p<0,001), muito rica (PR=0,81; 95%CI 0,75-0,86; p<0,001), e hipertensao (PR=0,91; 95%CI 0,867-0,969; p=0,002).

**Discussao:**

O sexo masculino foi o único fator associado com a nao realizagao de triagem para diabetes mellitus tipo 2, enquanto pertencia a uma faixa etária mais velha, ter hipertensao, educagao superior e status socioeconómico aumentou a probabilidade de triagem.

**Conclusao:**

É essencial reforgar as estratégias de triagem no nível da atengao primária através da implementagao de medidas preventivas.

## Introducción

La Diabetes Mellitus tipo 2 (DM2) es una enfermedad que representa un reto para la salud pública por su tendencia al crecimiento e impacto sobre todo en países en desarrollo^1^ . Según la Federación Internacional de Diabetes (IFD), en el año 2015, alrededor de 415 millones de personas en edad adulta en el mundo vivían con diabetes mellitus y se estima un incremento de esta cifra a 642 millones para el 2040^2^. Asimismo, en EE.UU., la prevalencia de DM2 oscila en un 10,5%;^3^ mientras que en el Perú se encuentra entre el 3 y el 7% ^4^^,^^5^.

Debido a que el impacto social y económico que genera esta enfermedad es importante, con un gasto de alrededor del 11,5% del gasto en salud para el 2021^6^ organizaciones como la Asociación Americana de Diabetes (ADA) plantean que debería abordarse este problema con un enfoque preventivo^7^, buscando métodos que no recurran a criterios laboratoriales^8^^,^^9^ o métodos en pasos secuenciales^10^. Dichos métodos han sido evaluados en estudios previos^11^. Además, en favor de lo anterior, se ha sugerido que estrategias de tamizaje temprano para DM2 pueden ser costo efectiva^12^, ayudar a reducir las complicaciones a largo plazo^13^, y reducir un posible daño para los pacientes^14^.

Si bien se han realizado trabajos a nivel internacional, sus resultados pueden diferir en nuestro medio, por las características encontradas, como la transición demográfica, inequidad social, cambios socioculturales, entre otros^15^^-^^22^ que son variables, aunque similares en Latinoamérica, diferentes entre cada país. En el Perú se ha observado un crecimiento exponencial de la diabetes, esta se estima en 20/1000 habitantes al año, cifras que siguen en aumento, más aún cuando existen hábitos alimenticios inadecuados dentro de la familia, esto estaría estrechamente relacionado a la cultura gastronómica, principalmente basada en el consumo de carbohidratos. Por ello, resulta importante conocer las características de los pacientes que se realizan cribado en el Perú, así como los factores que se encuentran asociados a esta conducta, además de poder contrastar esta información con datos de países latinoamericanos, los cuales comparten realidades similares, más aún en el contexto de la atención primaria de salud. Con ello, se podrían elaborar mejores protocolos y lineamientos que estén encaminados a la prevención y disminución de la incidencia de esta enfermedad^23^. En este sentido, el objetivo de este trabajo de investigación fue determinar los factores asociados a la no realización del cribado de diabetes mellitus a través del análisis de la ENDES-2020.

## Materiales y Métodos

### Diseño del estudio

Estudio observacional, analítico de corte transversal.

### Población y muestra

La muestra estuvo conformada por toda la base de datos de la Encuesta Demográfica y de Salud Familiar (ENDES) del año 2020, que corresponde a 37 390 viviendas a nivel urbano y rural. La muestra se caracterizó por ser bietápica, probabilística de tipo equilibrado, estratificada e independiente, a nivel departamental y por área urbana y rural. Por eso mismo, esta se caracteriza por ser representativa a nivel nacional y con un tamaño de muestra adecuado para asegurar una respuesta adecuada a los objetivos de investigación propuestos. La ENDES 2020 incluye una muestra de 32.197 hombres y mujeres de 15 años o más, de 25 regiones del Perú. Este estudio incluyó a las personas de 15 años a más que respondieron las preguntas del cuestionario de interés. Se excluyó a los pacientes con diagnóstico de diabetes y a quienes consumieran medicamentos que modifican los niveles de glucosa.

### Definición de variables

La variable respuesta fue la realización o no de cribado para DM2. Esto fue medido a través de la pregunta (QS107) "¿Le midieron el azúcar o glucosa en la sangre?". De esa manera se trabajó de manera dicotómica en sí vs no/no me acuerdo.

Se consideraron las siguientes variables como factores asociados: sexo (masculino, femenino), grupo de edad (categorizado en 15 a 29, 30 a 59, 60 años a más), área de procedencia (rural y urbano), nivel de educación (ninguno, primaria, secundaria, superior), nivel socioeconómico (muy pobre, pobre, medio, rico, muy rico), etnia (quechua, aymara, amazonía, nativo de otro pueblo, moreno, blanco, mestizo, otro), fumador activo (no, sí), consumo de alcohol (no, sí), índice de masa corporal (bajo peso, normopeso, sobrepeso y obesidad) e hipertensión arterial (HTA) (no y sí)

### Procedimiento

La ENDES-2020 se encuentra de libre acceso en la página web: https://www.inei.gob.pe/. Se accedió a la opción de base de datos, y de ahí al Sistema de Documentación Virtual de investigaciones Estadísticas. Luego, se ingresó a la sección de encuestas de hogares donde se seleccionó el apartado correspondiente. Se procedió a descargar las que se utilizan el estudio.

### Análisis estadístico

Se utilizó el software estadístico STATA 17. En el análisis univariado se procesó la información para el cálculo de frecuencias y porcentajes. Luego, se utilizó la regresión de Poisson con varianza robusta, que permitió calcular las razones de prevalencia (PR) crudas y ajustadas con sus respectivos intervalos de confianza. La selección de covariables en el análisis de regresión ha sido conforme a lo encontrado en la literatura previa. En la selección de análisis, se han utilizado los comandos "svy" y "svyset" para considerar los pesos muestrales en el estudio.

### Aspecto ético

Al ser un análisis secundario de datos libre, las observaciones se mantuvieron en el anonimato, por lo que los probables daños hacia los sujetos de estudio fueron mínimos. A su vez, este manuscrito fue aprobado por el comité de ética de la Facultad de Medicina de la Universidad Ricardo Palma (Código del Comité: PG 242-021)

## Resultados

Luego de la aplicación de los criterios de selección, en el presente trabajo se estudió a 20 497 sujetos. El sexo femenino estuvo conformado por el 51,38%. El 17,74% se encontró dentro del grupo de 60 años a más. Solo el 15,10% vivía en zona rural y el 1,01% no tenía educación alguna. La mitad se autoreportó mestizo (50,10%). El 16,87% era fumador y el 71,13% consumía bebidas alcohólicas. La prevalencia de HTA fue 22,46%. El 40,73% tenía sobrepeso. El 72,26% no se había realizado cribado para DM2. (Tabla 1).

**Tabla 1:** Características generales de la población estudiada de la ENDES, 2020

Características	n (% ponderada)¥
Sexo	
Femenino	11034 (51,38)
Masculino	9463 (48,62)
Grupo de edad	
15 a 29 años	5869 (30,39)
30 a 59 años	11182 (51,87)
60 años a más	3446 (17,74)
Área de residencia	
Rural	4525 (15,10)
Urbano	10699 (84,90)
Nivel de Educación	
Ninguno	224 (1,01)
Primaria	3020 (14,49)
Secundaria	6779 (44,54)
Superior	4875 (39,96)
Etnia	
Quechua	6050 (22,30)
Aymara	764 (1,84)
Amazonía	306 (0,83)
Nativo de otro pueblo	48 (0,18)
Moreno	2127 (11,29)
Blanco	1117 (6,70)
Mestizo	8786 (50,10)
Otro	1299 (6,77)
Nivel socioeconómico	
Muy pobre	3880 (13,38)
Pobre	3905 (19,32)
Medio	3151 (22,24)
Rico	2397 (21,98)
Muy rico	1861 (23,09)
Fumador activo	
No	17289 (83,13)
Si	3208 (16,87)
Consumo de Alcohol	
No	6475 (28,87)
Si	13557 (71,13)
IMC categorizado	
Bajo peso	213 (0,96)
Características	n (% ponderada)¥
Normopeso	6741 (31,26)
Sobrepeso	8344 (40,73)
Obesidad	5195 (27,05)
Hipertensión arterial	
No	16677 (77,54)
Si	3820 (22,46)
Se realizó cribado para diabetes	
No	15574 (72,26)
Si	4923 (27,74)

*¥Algunos valores no suman 20 497 debido a datos faltantes.*

Se realizó un análisis de regresión de Poisson considerando el peso muestral (Tabla 2). El análisis de regresión multivariable fue ajustado por sexo, grupo de edad, área de residencia, nivel de educación, etnia, nivel socioeconómico, fumador activo, consumo de alcohol, IMC e HTA. Los pacientes de sexo masculino tuvieron 1,06 veces la posibilidad de no realizarse una prueba de cribado para DM2, en comparación con el sexo femenino (PR=1,06, IC 95% 1,02 - 1,10; p<0,001). Los grupos de edad de 30 a 59 años y 60 años a más tuvieron un 8% (0,92; IC 95% 0,89 - 0,95; p< 0,001) y 28% menos (PR = 0,72; IC 95% 0,65 - 0,79; < 0,001) posibilidad de no realizarse una prueba de cribado para DM2, respectivamente, con relación al grupo de edad de 15 a 20 años. En relación con la educación, los que recibieron educación primaria, secundaria y superior mostraron 6% (PR=0,94, IC95% 0,92-0,99; p < 0,020), 7% (PR=0,93; IC 95% 0,88-0,97; p=0,008) y 14% menos (PR=0,86, IC 95% 0,85-0,94; p<0,001) posibilidad de no realizarse una prueba de cribado para DM2, respectivamente, con respecto a los que reportaron no haber recibido ningún tipo educación.

En caso de la riqueza, los que pertenecieron a los niveles socioeconómicos pobre, medio, rico y muy rico tuvieron 4% (PR=0,96, IC 95% 0,92-0,99; p=0,016), 7% (PR=0,93; IC95% 0,88-0,96; p=0,001), 11% (PR=0,89; IC 95% 0,84-0,94; p<0,001) y 19% menos (PR=0,81; IC95% 0,75-0,86; p<0,001) posibilidad de no realizarse una prueba de cribado para DM2, respectivamente, comparado con los de nivel socioeconómico muy pobre.

Finalmente, las personas con HTA tuvieron 9% menos posibilidad de no realizarse una prueba de cribado para DM2, a diferencia de los no hipertensos (PR=0,91; IC 95% 1,02 - 1,10; p=0,002).

**Tabla 2:** Análisis de regresión de Poisson crudo y ajustado de los factores asociados al cribado para DM2 según ENDES 2020

Características	Análisis Crudo			Análisis Ajustado*		
	RPc	(IC 95%)	^p^	RPa	(IC 95%)	^p^
Sexo						
Femenino	Ref.			Ref.		
Masculino	1,07	(1,04 - 1,10)	<0,001	1,06	(1,02 - 1,10)	0,001
Grupo de edad						
15 a 29 años	Ref.			Ref.		
30 a 59 años	0,87	(0,85 - 0,90)	<0,001	0,92	(0,89 - 0,95)	<0,001
	RPc	(IC 95%)	^p^	RPa	(IC 95%)	^p^
60 años a más	0,71	(0,67 - 0,74)	<0,001	0,72	(0,65 - 0,79)	<0,001
Área de residencia						
Rural	Ref.			Ref.		
Urbano	0,83	(0,81 - 0,86)	<0,001	0,97	(0,94 - 1,01)	0.113
Nivel de Educación						
Ninguno	Ref.			Ref.		
Primaria	0,90	(0,86 - 0,95)	<0,001	0,94	(0,92 - 0,99)	0,020
Secundaria	0,85	(0,81 - 0,89)	<0,001	0,93	(0,88 - 0,97)	0,008
Superior	0,71	(0,68 - 0,76)	<0,001	0,86	(0,85 - 0,94)	<0,001
Etnia						
Quechua	Ref.			Ref.		
Aymara	1,02	(0,93 - 1,10)	0,717	1,01	(0,94 - 1,09)	0,726
Amazonía	1,11	(0,99 - 1,25)	0,055	0,99	(0,89 - 1,12)	0,986
Nativo de otro pueblo	1,11	(0,86 - 1,42)	0,427	0,90	(062 - 1,29)	0,570
Moreno	0,99	(0,94 - 1,04)	0,765	0,90	(0,94 - 1,04)	0,690
Blanco	0,96	(0,90 - 1,02)	0,144	1,02	(0,96 - 1,09)	0,467
Mestizo	0,91	(0,88 - 0,94)	<0,001	0,97	(0,93 - 1,00)	0,080
Otro	0,97	(0,91 - 1,02)	0,273	0,99	(0,93 - 1,07)	0,852
Nivel socioeconómico						
Muy pobre	Ref.			Ref.		
Pobre	0,92	(0,89 -0,95)	<0,001	0,96	(0,92 - 0,99)	0,016
Medio	0,86	(0,82 - 0,88)	<0,001	0,93	(0,88 - 0,96)	0,001
Rico	0,81	(0,77 - 0,84)	<0,001	0,89	(0,84 - 0,94)	<0,001
Muy rico	0,70	(0,66 - 0,74)	<0,001	0,81	(0,75 - 0,86)	<0,001
Fumador activo						
No	Ref.			Ref.		
Si	1,02	(0,98 - 1,06)	0,300	0,97	(0,93 - 1,02)	0,216
Consumo de Alcohol						
No	Ref.			Ref.		
Si	1,02	(0,98 - 1,05)	0,198	1,08	(1,01 - 1,05)	0,345
IMC categorizado						
Bajo peso	Ref.			Ref.		
Normopeso	0,94	(0,85 - 1,04)	0,228	0,96	(0,86 - 1,08)	0,518
Sobrepeso	0,84	(0,76 - 0,92)	<0,001	0,93	(0,82 - 1,04)	0,188
Obesidad	0,80	(0,73 - 0,88)	<0,001	0,91	(0,81 - 1,02)	0,117
Hipertensión arterial						
No	Ref.			Ref.		
Si	0,80	(0,77 - 0,83)	<0,001	0,91	*(0,867 -* 0,969)	0,002

*cada variable ha sido ajustada por las otras presentes en la tabla 2.*Valorp significativo <0,05 RP: Razón de prevalencias. IC 95%: Intervalo de confianza al 95%

## Discusión

### Hallazgos principales

De los hallazgos estadísticamente significativos, la única variable que disminuyó la posibilidad de no realizarse el cribado fue pertenecer al sexo masculino, mientras que pertenecer a un grupo de edad menor, presentar HTA, tener un mayor nivel de educación y socioeconómico aumentó la posibilidad de realizarse este.

### Comparación con otros estudios

Pertenecer al sexo masculino fue un factor que aumentó la posibilidad de no realizarse el tamizaje de DM2. Estos resultados son concordantes con diferentes estudios que han reportado que en particular el sexo masculino muestra tanto menores tasas de realización de cribado para DM2^20^^,^^22^^,^^24^^,^^25^, así como mayores conductas de salud de riesgo comparadas con el sexo femenino^26^.

Esta discrepancia entre sexos puede ser potencialmente explicada considerando que tal como se ha reportado, las mujeres expresan mayores niveles de interocepción^27^^,^^28^, lo que se relaciona con una mayor disposición y capacidad para cuidar de sí mismas cuando están enfermas y buscar atención de forma preventiva más frecuentemente que el varón; adicionalmente, se ha reportado que existen varios comportamientos masculinos estereotípicos que predisponen a los varones a estar menos dispuestos a buscar consejo médico^26^, ya que la importancia que estos le dan a la autosuficiencia, fortaleza física y control emocional inhiben su disposición a buscar ayuda^29^. Esto, sumado a la negación, la vergüenza y el deseo de evitar una situación en la que no tienen el control, favorece a que tiendan a hacerse menos exámenes físicos anuales^24^^,^^30^.

El pertenecer a un grupo de edad mayor aumentó la posibilidad de realizarse un cribado, en particular en el grupo de 60 años a más. Resultados similares han sido reportados previamente en distintos estudios^19^^,^^22^^,^^24^^,^^31^, lo que indicaría que existen disparidades entre grupos de edad para la realización de cribados de forma preventiva. Esto cobra trascendencia cuando se considera que la edad recomendada para tamizaje según las guías de la ADA, ha pasado de ser 45 años a 35 años^9^, además que la detección de grupos más jóvenes y de alto riesgo puede ser más rentable que la detección de personas mayores^32^ lo que puede ofrecer un beneficio para la salud pública.

Por otro lado, las diferencias entre grupos de edad podrían estar relacionadas con el hecho de que suele existir una mayor participación de grupos mayores de edad en servicios de salud^19^^,^^24^, tanto por el hecho de que esta es un factor de riesgo en sí misma para diferentes enfermedades crónicas como la DM2^9^, así como por las diferencias en la posibilidad de contar con cobertura de aseguramiento de salud las cuales aumentan con la edad^33^. De hecho, otro hallazgo del presente estudio fue que un mayor nivel socioeconómico se asoció con la mayor posibilidad de haber realizado el tamizaje para DM2, lo que tal como se ha encontrado en otros trabajos^19^^,^^22^^,^^24^^,^^31^^,^^34^, refleja un patrón global respecto al acceso a servicios de salud^35^, mismos que estarían limitados a las personas de menores ingresos.

En el caso particular de Perú, aunque la cobertura de aseguramiento de salud ha mostrado un crecimiento en el periodo 2009-2017, se mantienen brechas entre el crecimiento subsidiado (SIS) y el contributivo (ESSALUD), con un mayor crecimiento del segundo en el quintil más alto de riqueza^33^, mismo que concentra el mayor volumen de atención de enfermedades crónicas^36^.

Un mayor nivel educativo aumentó la posibilidad de realizarse una prueba de cribado para DM2. Esto concuerda con estudios previos^19^^,^^22^^,^^24^^,^^34^^,^^37^, en los que estos factores se reportaron como potenciales barreras a la realización de tamizaje cuando los participantes tuvieron menor nivel educativo, principalmente por un menor entendimiento del procedimiento y propósito del mismo^22^. Adicionalmente, en otras condiciones crónicas el bajo nivel educativo se ha relacionado también con una menor percepción del riesgo de la enfermedad^38^^,^^39^, lo que potencialmente podría explicar los resultados del presente estudio. Por otro lado, en el Perú se ha documentado que un menor nivel educativo disminuye la posibilidad de contar con cobertura de aseguramiento de salud de tipo contributivo (ESSALUD)^33^, lo que podría explicar el menor acceso a servicios especializados en tratamiento y prevención de enfermedades crónicas.

Debe resaltarse que aunque algunos grupos se encuentren menos informada sobre las pruebas de cribado de DM2 y otras enfermedades^16^^,^^18^, realizar el cribado en esta zona es factible^15^, por lo que cerrar las brechas existentes entre estas áreas de residencia, resulta importante para la salud pública.

Los hallazgos del presente estudio no respaldan la existencia de una asociación entre la etnia y la no realización del cribado para DM2. Sin embargo, estos resultados contrastan con lo encontrado por otros autores^19^^,^^34^^,^^35^, quienes reportaron que las minorías étnicas tuvieron menor posibilidad de realizarse el cribado, mientras que en particular las personas de ascendencia afroamericana tuvieron una mayor predisposición a realizárselo. Por otro lado, aun cuando se ajusta por factores sociodemográficos y clínicos, la asociación entre etnicidad y realización del cribado se mantuvo en un estudio que trabajó con población de Estados Unidos^21^.

La presencia de HTA aumentó la posibilidad de hacerse un cribado entre los participantes de nuestro estudio. Resultados similares han sido previamente reportados tanto para la presencia de HTA como de otras comorbilidades como factores que predisponen a las personas que las padecen a realizarse un tamizaje para DM2^19^^,^^24^^,^^25^^,^^31^. Estos resultados sugieren que el patrón de realización de tamizaje responde a la presencia de mayor riesgo asociado en las personas que lo padecen. En respaldo de lo anterior, se ha documentado que el nivel de concientización hacia DM2 y su tamizaje aumenta cuando se padece de otra comorbilidad, posiblemente por la experiencia previa que se tiene sobre lo que implica vivir con una enfermedad crónica^40^.

Por último, ser fumador, consumir bebidas alcohólicas y el IMC, no presentaron asociación con la realización del tamizaje. Resalta en particular este último hallazgo ya que en la literatura científica niveles de IMC concordantes con el sobrepeso y/o obesidad aumentan la posibilidad de realizarse el cribado^17^^,^^19^^,^^24^^,^^25^^,^^40^. Probablemente, este resultado se manifestó porque tanto ser fumador, consumir bebidas alcohólicas, o padecer de sobre peso u obesidad, no son considerados como una enfermedad en el Perú por distintos factores culturales que a su vez varían entre las diferentes regiones^41^^-^^44^. Es decir, hay una normalización de estas condiciones que hace que la población no tenga el nivel de concienciación que les permita reflexionar sobre las consecuencias de estas condiciones para su salud en un futuro^44^.

### Limitaciones del estudio

Esta investigación tiene tanto fortalezas como limitaciones. Primero, al ser un estudio transversal, no puede determinarse causalidad; no obstante, esto puede ser un primer alcance del comportamiento de estas variables para la no realización de cribado en nuestro medio. En segundo lugar, este es un análisis secundario de una base de datos recolectada para otro objetivo, por lo que en futuros estudios para confirmar estos resultados se recomienda hacer una recolección primaria.

## Conclusiones

De los hallazgos estadísticamente significativos, la única variable que disminuyó la posibilidad de no realizarse el cribado fue pertenecer al sexo masculino, mientras que pertenecer a un grupo de edad menor, presentar hipertensión arterial, tener un mayor nivel de educación y socioeconómico aumentó la posibilidad de realizarse este. De confirmarse esto en futuros estudios, se debe prestar atención a estas características para aumentar la frecuencia de tamizaje de esta enfermedad en nuestro medio, más aún en nivel primario de atención.
